# The British Red Cross Society and the Territorial General Hospitals

**Published:** 1908-07-11

**Authors:** 


### The Royal Army Medical Corps Section.

### THE BRITISH RED CROSS SOCIETY AND THE TERRITORIAL GENERAL HOSPITALS.

Since its inauguration at a meeting held at Buckingham Palace on July 17, 1905, under the presidency of her Majesty the Queen, the British Red Cross Society has been very properly engaged in establishing an organisation which will be uniform throughout the United Kingdom. The shape this organisation has taken is that of committees in each county and in all the large cities and towns. Everywhere there has been a ready response on the part of those asked to join in this Red Cross movement, and the last three years have seen the establishment of a great many excellent committees that we are sure will be quite ready, if called upon in time of war or invasion, to organise and deal with the offers of voluntary aid made in their districts, thus preventing that overlapping of effort which is such a fertile source of waste, not only of time, but of money and material, and leads to endless confusion.

Although such committees are officially termed *latent*, they are empowered to concern themselves actively with Red Cross work in peace time should any occasion arise.

An excellent opportunity for their activity has arisen in connection with the establishment of the General Hospitals of the Territorial Army. In this matter the Red Cross Committees of those large towns where those hospitals are located can give valuable help in a way that may add materially to their usefulness and efficiency. As we have already pointed out, these hospitals are to exist in a skeleton shape; a condition associated always with the danger that they may exist only on paper, so that when called up they are found to be quite unserviceable. An influential and active Red Cross Committee could in time of peace take steps to prevent such an unfortunate result, and could insist on their being made a reality and properly provided for.

Chief amongst the matters that should be previously arranged for in connection with these Territorial General Hospitals are the provision of (*a*) proper quarters and (*b*) equipment. The former is a point of no small importance, and one that requires very full consideration. The aggregation of a large number of sick and wounded persons in a common building has its attendant risks and dangers, unless that building has the cubic space and the sanitary arrangements necessary. In every town where a General Hospital is to be located it should be settled beforehand that such accommodation exists, and that it will be available at once when required. Further, all the required alteration should be decided on and arrangements made for carrying them out at once. On the other hand, should it happen, as it well may, that a suitable building does not exist, then a decision should be come to as to a site for a temporary hospital and as to the form this latter should take. The selection of a site is not the least important of these two questions, for it involves attention to soil, proximity and purity of water-supply, the practicability of drainage, the shelter afforded from cold winds by wood or high land, and the accessibility of the position both as regards the railway and the main road. As to the form such a temporary hospital should take, the choice is limited to tents, or to Doecker huts, or to those made of galvanised iron, wood, or Willesden waterproof material. In summer tents would probably be best, while in winter the wooden, Willesden, or Doecker huts would be preferable, all of them, too, being easier to warm than those of galvanised iron. Whichever be decided on, it is imperative that provision be made for their immediate supply when needed. An assurance of a very definite kind should be obtained from the military authorities that they would be at once forthcoming, or the local Red Cross Committee should obtain powers to arrange for their purchase at Government expense. The same remarks apply equally to the equipment and furnishings of the hospitals. While these should be reduced to a minimum, it is very important that all the medical stores, bedding, clothing, and furniture necessary for the working of the hospital should be procurable at very short notice, and should be serviceable.

Another important detail that might very well engage the attention of the local Red Cross Committees is the provision of that portion of the hospital staff which is needed to bring it up to its full strength when the hospital is mobilised. Seeing that the female nurses are to be provided for by a special arrangement, the task of procuring the balance of the *personnel* is one where local knowledge would be invaluable, for by it there could be compiled a list of reliable persons willing to come forward and help in the hospital in time of war or invasion. To those enrolling some instruction might have to be given, but it would not be of an elaborate kind, as the duties assigned them would not demand a very special training.

Besides the above there are several other matters that might very well be dealt with by Red Cross Committees, such as the transport of the sick and wounded between the field ambulances and the General Hospitals, the establishment of refreshment stations along the lines of communication and the provision of convalescent homes for officers and men. The last two items have not the same immediate urgency as the first. For this latter there should exist some well-devised and pre-arranged scheme. One has only to contemplate the confusion that would arise, and the suffering that might be inflicted on the wounded after an engagement if there was not in existence some previously prepared plan for their proper removal by road, rail, or water. It would not be creditable to us as a nation if all this had to be organised when the demand arose, instead of having everything ready, as should be the case, for an emergency that might possibly arise and consequently should be provided for.

In view of this opinion that our local Red Cross Committees can be of considerable service to the Territorial General Hospitals, we think that it is a matter of congratulation that negotiations have taken place and an agreement come to between the Army Council and the British Red Cross Society in connection with all the points mentioned above. Under this agreement the Society will make use of the machinery of its county and other committees to render assistance to the hospitals in these matters, and a special Memorandum has very properly been issued by the Army Council to the various County Associations urging them to come into touch with the County and other Committees of the British Red Cross Society with the object of co-operation in this important work. We sincerely hope that this conjoined action will take place. In the first place, it will be in the interests of the General Hospitals that it should. Under the combined influence of the British Red Cross Society and the County Associations these hospitals may be made a living reality, and not exist as a mere phantom creation of the official mind. In the next place, we consider that this arrangement will benefit the British Red Cross Society, for it will bring the latter more into touch with the people generally than has been the case in the past. In fact, such a relationship between the Society and the County Associations is to some extent the realisation of the hopes we expressed as far back as July 1905, when, on the inauguration of the Society, we indicated that its aim should be to establish in each county, after conference with the Army Medical Department, a hospital fully equipped for active service.

